# Blockchain in Healthcare: A Patient-Centered Model

**Published:** 2019-08-08

**Authors:** Hannah S Chen, Juliet T Jarrell, Kristy A Carpenter, David S Cohen, Xudong Huang

**Affiliations:** Department of Psychiatry, Massachusetts General Hospital and Harvard Medical School, USA

**Keywords:** Blockchain, Data Security and Confidentiality, E-health, Electronic Health Records, Healthcare Service Innovation and IT, Information and Knowledge Management, IT Design and Development Methodologies, Telehealth

## Abstract

A blockchain is a system for storing and sharing information that is secure because of its transparency. Each block in the chain is both its own independent unit containing its own information, and a dependent link in the collective chain, and this duality creates a network regulated by participants who store and share the information, rather than a third party. Blockchain has many applications in healthcare, and can improve mobile health applications, monitoring devices, sharing and storing of electronic medical records, clinical trial data, and insurance information storage. Research about blockchain and healthcare is currently limited, but blockchain is on the brink of transforming the healthcare system; through its decentralized principles, blockchain can improve accessibility and security of patient information, and can therefore overturn the healthcare hierarchy and build a new system in which patients manage their own care.

## Introduction

In the world of healthcare today, there are two major focuses that must be addressed: data security and data ownership. Sensitive medical records currently lack a secure structure, leading to data breaches with severe consequences. In 2018, the Department of Health and Human Services’ Office for Civil Rights (OCR) received notifications of many data breaches that resulted in the exposure of 13 million total healthcare records [1]. Furthermore, according to a recent study conducted by the Ponemon Institute on behalf of IBM Security, the average total cost of data breach in the United States was $7.91 million, with the health sector having the highest per capita cost (Ponemon Institute, 2018). Another concern is that patients are currently unable to have full ownership of their own medical data, a notion that is increasing in relevance with the rise of personalized medicine and wearables. Both these issues also lead to significant moral repercussions that must be resolved.

Blockchain technology may provide the answer.

**Figure 1:**
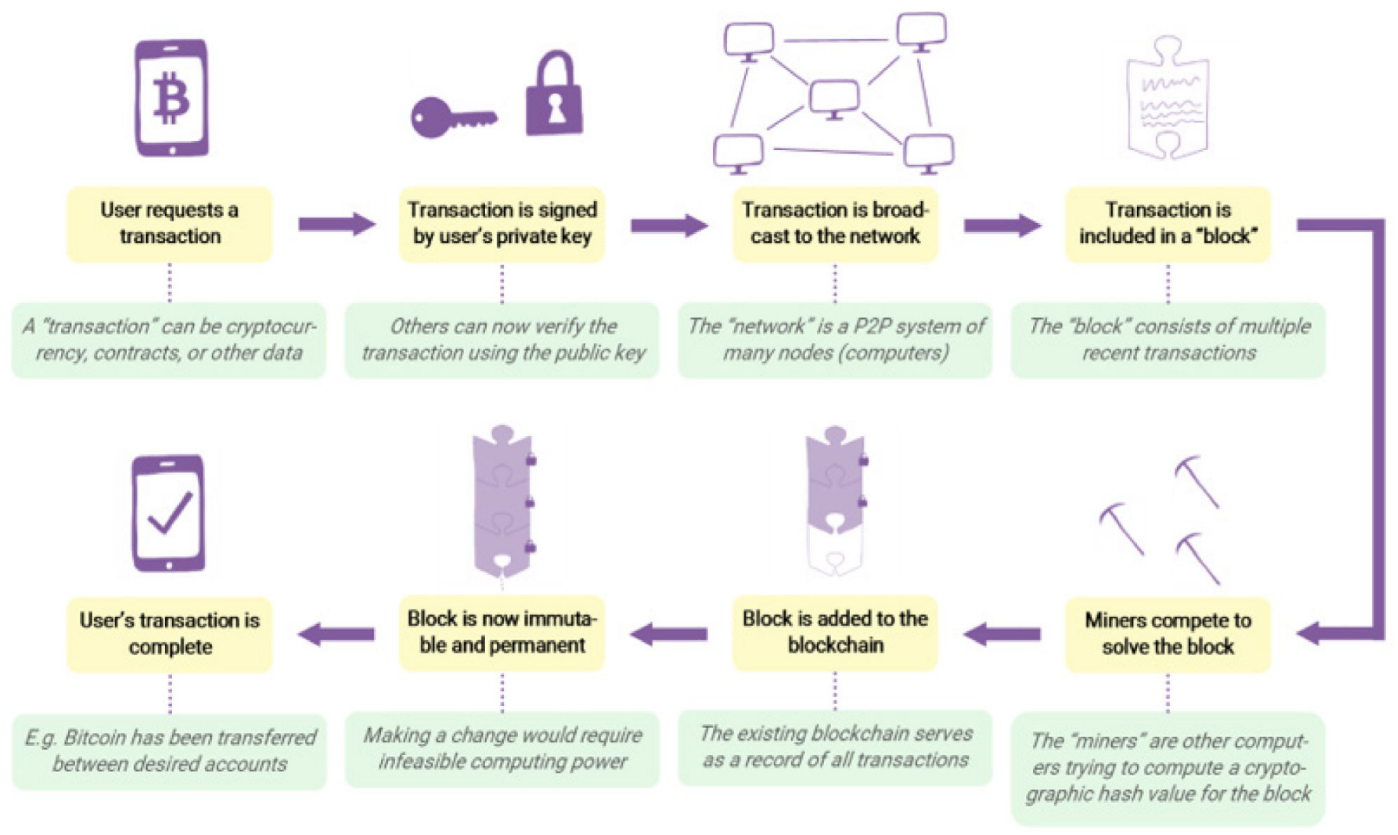
Overview of how a blockchain system operates.

Invented in 2008 by Satoshi Nakomoto for the cryptocurrency Bitcoin, the technology offers a verifiable, permanent, and attack-resistant system for recording data [2]. Blockchain is a decentralized, distributed digital ledger that records transactions in a growing chain of immutable blocks linked by cryptographic hashes. Figure 1 provides an overview of how a blockchain system, such as the original Bitcoin, carries out functions. A user begins by requesting a transaction (a transaction may involve cryptocurrency, contracts, records, or other data). The user signs the transaction with his private key, which allows others to verify the authenticity of this transaction using the public key (a public key is generated from the private key through a one-way mathematical function, commonly elliptic curve multiplication) [3]. Next, the transaction is broadcast to the entire peer-to-peer network of nodes, and miners each select a batch of transactions to form a block. (Note that a miner is always a full node, but a node is not necessarily a miner.) Each miner now competes to solve for an accepted hash output of the transactions encoded in his block.

This process involves using computation power to test random input strings until one produces an output string that meets the requirements. The first miner to succeed gets to add his block to the blockchain, thus completing the transaction, and the next round begins (Figure 1). Blockchain is permanent because once a block is added to the chain, anyone who wishes to alter it would need to recompute the altered block and all subsequent blocks, which requires an infeasible amount of computation power. Furthermore, blockchain is secure because there is no centralized structure for a malicious user to target, as the data is stored in numerous copies on different nodes. These properties render blockchain ideal in healthcare data management. Although the application of blockchain to healthcare is a relatively new exploit, more research is becoming available each day. Booz Allen Hamilton Inc. has begun a pilot project for blockchain use in the hospital setting (Cyran), and various mobile apps and Electronic Medical Record (EMR) systems based on blockchain have also been proposed. Table 1 provides a summary of these systems.

## Mobile Health and Remote Monitoring

Mobile applications and remote monitoring machines are essential for patient care in today’s technological age. Blockchain can be applied to these technologies and improve security and quality of machinery. For example, a group of researchers created and tested an mHealth (mobile health) smart phone application for cognitive behavioral therapy for insomnia [4]. The application sends patient health data to a private blockchain network. The EMRs in the network were secure and resistant to tampering after testing because of the properties of the blockchain, and the data was accessible to and controlled by the patient. Using an application such as this one, the patient can record data and send it to healthcare providers in minutes no matter the distance between the patient and the provider. Since patients can monitor their own care using this system, patients may be more attentive to their own health and well-being.

Furthermore, another mobile application, called Healthcare Data Gateway (HGD), has been presented for organization of patient data [5]. The application uses a simple unified Indicator-Centric Schema (ICS) to organize this data, and a secure Multi-Party Computing (MPC) system, which enables third party interaction with protected healthcare data without privacy breaches and with-out authoritative power over the information. This application houses different layers of data: the storage layer, data management layer, and the data usage layer, that work together to enforce proper security and functionality of information. Additionally, improving blockchain based smart contracts may help monitoring devices operate securely. Smart contracts verify and monitor the blockchain activity. A private blockchain which is based on the Ethereum model has been developed to improve remote monitoring; in this private blockchain, sensors interact with smart devices that utilize smart contracts to record events in the blockchain [6]. The smart contracts enable real-time patient monitoring, as important notifications are sent to patients and healthcare providers securely. Real-time updates are essential for safe care at home and enable patients to take charge of their own care while a healthcare provider is always accessible.

Security concerns are associated with mobile monitoring of healthcare, as patient health information is private and sensitive. Mobile malware has destroyed blockchain in the past, and the blockchain that controls mobile applications must be completely secure before it can be used to store health information. One study investigated root exploit, the most vicious type of mobile malware, and its interactions with blockchain [7]. Root exploit is dangerous to blockchain systems because it can obtain a patient’s PKI private key that is used by the patient for access to their personal, protected information in the blockchain. The biological method of Practical Swarm Optimization (PSO) was used in this study to select for root exploits, along with boosters, which can strengthen machine learning activity to uncover new root exploits and glean new information about malware. Logitboost, a type of boost used in the study, was found to detect root exploit at a rate of 93% accuracy.

Moreover, Logitboost was also found to predict root exploit in the Root Exploit Detection System (RODS) curated in the study. More studies need to investigate mobile health and remote monitoring security concerns to ensure that the blockchain can store patient information successfully within these types of devices.

## Accessing and Sharing Health Data

Blockchain technology can also be applied to the accessing and sharing of patient medical records. Medical records can be difficult to access because they are generally spread out across many different healthcare facilities, and blockchain can enable patients to have full and secure access to all of their records and medical history. A few systems based on blockchain have been proposed for organization of and access to patient medical records. MedBlock, a blockchain based information management system, enables efficient EMR access and retrieval through distributed blockchain principles [8]. The improved consensus mechanism of this software ensures that the network is not overloaded by activity. Network overload is a concern when software such as blockchain that continues to grow over time is implemented. Moreover, MedBlock is highly secured by access control and cryptography.

Similarly, a blockchain based Data Preservation System (DPS) for medical data has been engineered [9]. DPS uses similar data storage mechanisms and cryptographic algorithms to ensure security. A prototype of DPS has been created which is modeled after the Ethereum blockchain. Furthermore, one system, called omniPHR, places all patient health records (PHR) in one accessible place by incorporating different data sets into different blocks on the blockchain [10]. Each block is then encrypted and the chain of blocks carrying the different pieces of one patient’s health information is fully accessible to that patient. Patients should be able to access all of their medical data in one forum, and this access will improve efficiency of care. Another electronic health record (EHR) system has been established in addition to omniPHR, and this system combines attribute-based encryption (ABE), identity-based encryption (IBE), and identity-based signatures (IBS) to create a new type of cryptographic framework called combined attribute-based/identity-based encryption and signature (C-AB-/IB-ES) [11].

This new cryptographic framework based on multiple levels of encryption maximizes security. Sharing of healthcare data in a secure way is also an integral part of healthcare. Zhang and Lin curated a patient information sharing plan, called a BSPP (blockchain-based secure and privacy-preserving patient health information sharing) scheme, that may improve diagnosis of patients in virtual health systems [12]. Their scheme includes the use of private and consortium blockchains and consensus mechanisms for the utmost security. The private patient health information is stored in the private blockchain while the records of patient health information activity, rather than the actual information, are stored in the consortium blockchain, and all the data is encrypted.

Likewise, Dubovitskaya et al. [13] created an EMR sharing system for cancer patients that utilizes blockchain [13]. Their method emphasizes security, availability, privacy, and control of data access, and lowers access time for records. Another data sharing system was developed that uses LifePound, a digital currency that is similar to bitcoin, which can buy and sell biomedical data [14]. LifePound rewards a patient for entering data and encourages participation in data sharing. A patient’s location is another piece of health information that may need to be shared securely with providers and other relevant personnel. A Blockchain-Based Multi-Level Privacy Preserving Location Sharing scheme (BMPLS) has been proposed for telecare medical information systems [15]. Further research is needed to improve methods of patient information sharing.

Blockchain technology has also been applied to clinical trials and, very recently, to medical insurance storage. Blockchains can easily detect fake information because of their transparency, and smart contracts in the blockchain can serve as security promoters for clinical trial data [16]. These smart contracts can keep a truthful record of trials and produce truthful data that can fuel new and effective treatments and pharmaceuticals. Another study also outlines the benefits of blockchain in clinical trials and presents a successful procedure for implementation [17]. In addition to transforming clinical trials, blockchain could also transform medical insurance storage. A recent study outlines MIStore, a blockchain-based medical insurance storage system [18]. The storage system utilizes different hospital, patient, and insurance company servers that verify each other’s activity and security. Systems such as MIStore can encourage a more productive relationship between patients, caregivers, and insurance companies. This study is the only one of its kind so far, but blockchain has many potential applications for insurance and more research will certainly become available soon (Table 1).

## Proposed Patient-Centered Blockchain Model

**Figure 2:**
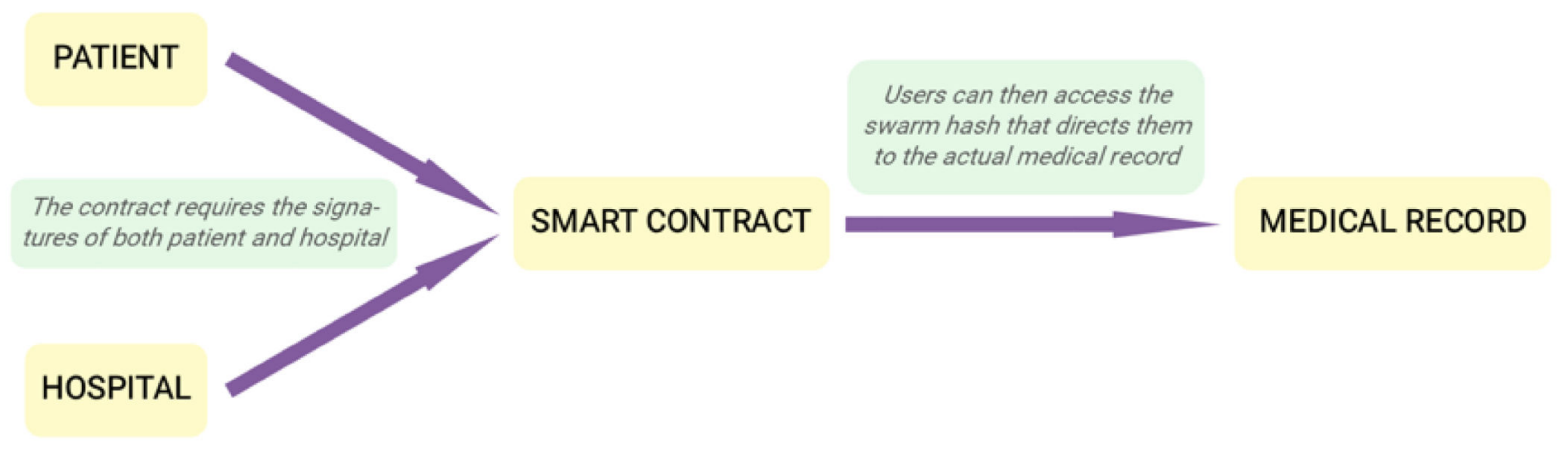
Overview of a proposed blockchain healthcare model.

We propose a medical data management system using blockchain technology that is secure and allows patients to retain ownership over their own records while allowing hospitals to have easy access (see the following Figure 2). We base our system on the Ethereum service, a decentralized platform that allows developers to run applications on a custom-built blockchain (Ethereum Foundation, 2018). Since many times, blockchains do not innately offer sufficient storage, we store the actual medical records on a decentralized cloud storage such as Ethereum Swarm, a native base layer service of the Ethereum web3 stack that functions as a distributed storage platform (Swarm, 2018). Each medical record then has a unique swarm hash, which combined with the decryption key, form the root chunk. Only those that know the reference to the root chunk can access the content. Thus, the root chunks are securely stored in smart contracts through the blockchain and are released only under specific conditions.

To solve the problem of data ownership and control, we employ multi-signature (multisig) contracts. Multisig requires multiple users, in this case the patient and the hospital, to both use their private keys to sign a transaction for authentication. This way, the patient cannot alter the record without permission of the hospital, but he still has control over who can access his record. A new swarm hash must be generated each time after the data has been accessed (since the old swarm hash is now known), so we add a “last accessed” timestamp. The change in the data will automatically change the swarm hash, which can then be secured once again until it receives the required permissions for access. This model not only provides the security and immutability provided by blockchain, but also offers a multisig solution to data ownership and accessibility.

## Addressed Concerns

Although blockchains are extremely secure, they are by nature susceptible to a type of attack known as the 51% attack (also called a double-spend attack or majority attack) (Yli-Huumo, Ko, Choi, Park, & Smolander, 2016). This occurs when a miner, or group of miners, controls over 50% of the total mining hash rate (computing power) in the network and becomes able to prevent new transactions or reverse completed transactions. By controlling over 50% of the computing power, the malicious user is able to form blocks at a faster rate than the rest of the network, and by the longest chain rule (which states that the longest blockchain is the valid chain), the network is forced to switch to the attacker’s desired chain. However, although 51% attacks are possible, the probability of a successful attack is extremely low, so blockchain remains one of the most secure forms of technology.

Another potential concern is the cost of implementing a blockchain system. When a transaction is requested, the user must pay for the computation. In the Ethereum network, the payment is calculated in “gas” and the gas is paid in “ETH,” valued around $120 USD as of January 2019 (EthereumPrice.org, 2019). The vast amount of transactions needed to be processed in hospital settings would amount to a sizable cost. However, the use of a blockchain system would replace current storage systems (electronic health records (EHRs), personal medical records, disease registries, and other databases (The National Academies)) and eliminate costly data breaches and other errors, ultimately rendering the system cost-efficient.

## Conclusion

Because blockchain maximizes security and accessibility, the technology can be used in many different areas of the healthcare system, such as for storing and sharing medical records and insurance information both in healthcare venues and in mobile applications and remote monitoring systems, and for clinical trials. Research about blockchain’s applications to healthcare is currently limited, but more research becomes available every day. Blockchain is one of the most active areas of software research currently, and it can change the hierarchy of healthcare by returning authority over medical records and health data to the patient. This transfer of authority may lead to an overall shift toward patient-centered care; the blockchain movement for patients is just beginning.

## Figures and Tables

**Table 1:** A summary of the reviewed blockchain systems in healthcare.

Name	Use	Key Features
Health application	Mobile application	-developed for cognitive behavioral therapy for insomnia
		-patient records own data and healthcare provider can access this data within minutes
Healthcare Data Gateway	Mobile application	-uses a simple unified indicator-centric schema (ICS) to organize data and a secure multi-party computing (MPC) system
Not named, Griggs et al. model	Remote monitoring	-sends secure real-time notifications to patients and healthcare providers via sensors that interact with smart devices which use smart contracts
Logitboost	Boosting algorithm	-can detect root exploit, a type of mobile malware that can destroy mobile health applications, at a rate of 93% accuracy
		-can predict root exploit in a simulated root exploit detection system
MedBlock	Electronic medical record (EMR) access system	-improved consensus mechanism ensures that network is not overloaded
Blockchain based data preservation system (DPS)	Storing medical data	-prototype modeled after Ethereum system has been tested
OmniPHR	Patient health records (PHR) access and storage system	-places all patient health data into one accessible forum
		-incorporates different patient datasets into different blocks on the chain
Not named, Wang and Song model	Electronic health record (EHR) storage and access system	-uses attribute-based encryption (ABE), identity-based encryption (IBE), and identity-based signatures (IBS) to create a type of cryptography called combined-attribute-based/identity-based encryption and signature (C-AB-/IB-ES) that maximizes security
Blockchain-based secure and privacy-preserving patient health information sharing (BSPP) scheme	Sharing of health data	-uses private blockchain for patient data and semi-private/consensus blockchain for non-sensitive, activity data
Not named, Dubovitskaya et al. model	Electronic medical record (EMR) sharing system	-developed for cancer patients
		-lowers access time for records
Not named, Mamoshina et al. model	Sharing of health data	-uses LifePound, a digital currency that can buy and sell biomedical data
Blockchain-based multi-level privacy preserving location sharing (BMPLS) scheme	Sharing of health data	-enables sharing of patient location
		-proposed for telecare medical information systems
MIStore	Medical insurance storage system	-uses different hospital, patient, and insurance servers that verify each other’s activity for security
